# Maternal High-Fat Diet Modulates Hepatic Glucose, Lipid Homeostasis and Gene Expression in the PPAR Pathway in the Early Life of Offspring

**DOI:** 10.3390/ijms150914967

**Published:** 2014-08-25

**Authors:** Jia Zheng, Xinhua Xiao, Qian Zhang, Miao Yu, Jianping Xu, Zhixin Wang

**Affiliations:** Department of Endocrinology, Key Laboratory of Endocrinology, Ministry of Health, Peking Union Medical College Hospital, Diabetes Research Center of Chinese Academy of Medical Sciences & Peking Union Medical College, Beijing 100730, China; E-Mails: zhengjiapumc@163.com (J.Z.); rubiacordifolia@yahoo.com (Q.Z.); yumiao@medmail.com.cn (M.Y.); jpxuxh@163.com (J.X.); wangzhixin-903@163.com (Z.W.)

**Keywords:** maternal high-fat diet, peroxisome proliferator-activated receptor, glucose and lipid homeostasis, weaning, offspring

## Abstract

Maternal dietary modifications determine the susceptibility to metabolic diseases in adult life. However, whether maternal high-fat feeding can modulate glucose and lipid metabolism in the early life of offspring is less understood. Furthermore, we explored the underlying mechanisms that influence the phenotype. Using C57BL/6J mice, we examined the effects on the offspring at weaning from dams fed with a high-fat diet or normal chow diet throughout pregnancy and lactation. Gene array experiments and quantitative real-time PCR were performed in the liver tissues of the offspring mice. The offspring of the dams fed the high-fat diet had a heavier body weight, impaired glucose tolerance, decreased insulin sensitivity, increased serum cholesterol and hepatic steatosis at weaning. Bioinformatic analyses indicated that all differentially expressed genes of the offspring between the two groups were mapped to nine pathways. Genes in the peroxisome proliferator-activated receptor (PPAR) signaling pathway were verified by quantitative real-time PCR and these genes were significantly up-regulated in the high-fat diet offspring. A maternal high-fat diet during pregnancy and lactation can modulate hepatic glucose, lipid homeostasis, and gene expression in the PPAR signaling in the early life of offspring, and our results suggested that potential mechanisms that influences this phenotype may be related partially to up-regulate some gene expression in the PPAR signalling pathway.

## 1. Introduction

Maternal nutrition has historically been a key determinant for offspring health, and gestation is the critical time window that can affect the growth and development of offspring. The developmental origins of health and disease (DOHaD) hypothesis proposes that exposures during early life play a critical role in determining the risk of developing metabolic diseases in adulthood [1]. Currently, there are substantial epidemiological studies and experimental animal models that have demonstrated nutritional disturbances during the critical periods of early life development can significantly impact the predisposition to developing adverse health outcomes in later life [2,3,4,5].

It has been widely accepted that undernutrition during early life increases the risk for intrauterine growth restriction and metabolic disease in adult life [6]. The incidence of obesity during pregnancy and gestational diabetes mellitus have recently been rapidly escalating, and the global prevalence of hyperglycaemia in pregnant women (20–49 years) is 16.9% [7]. Therefore, an increased number of studies have focused on maternal overnutrition during pregnancy; these studies indicated a maternal high fat diet can contribute to obesity, impaired glucose tolerance and dyslipidemia in offspring [8]. However, most feeding studies have utilized animal models that examined long-term paradigms. For example, a maternal high-fat diet is provided before pregnancy and during gestation and lactation periods, and the offspring are continuously fed a high-fat diet from young adulthood (post-weaning) to various durations that range from 8 to 36 weeks [3,9,10,11]. As a consequence, these studies were confined to long-term modifications in the maternal and offspring’s diets. Thus, the phenotype would be interfered by providing the offspring with a high-fat diet post weaning. Moreover, the consumption of a high-fat diet only during pregnancy and suckling is very common in humans. This issue is particularly important in light of the increasingly greater consumption of refined foods and the concerns regarding the critical periods of gestation and the suckling period.

However, whether maternal high-fat feeding can impact glucose and lipid metabolism during the early life of offspring is not well understood. We hypothesized that a maternal high-fat diet only during pregnancy and lactation may contribute to the development of obesity and the metabolic syndrome in the early life of the offspring at weaning. Furthermore, the mechanistic link between the maternal high-fat diet and the metabolic diseases of their offspring is not completely understood. Therefore, we explored the underlying mechanisms that influence the phenotype without the interference of the maternal diet condition before pregnancy or the offspring’s diet condition post weaning.

## 2. Results

### 2.1. Offspring of the Dams Fed a High-Fat Diet Had a Lower Birth Weight and a Heavier Body Weight at Weaning

**Figure 1:**
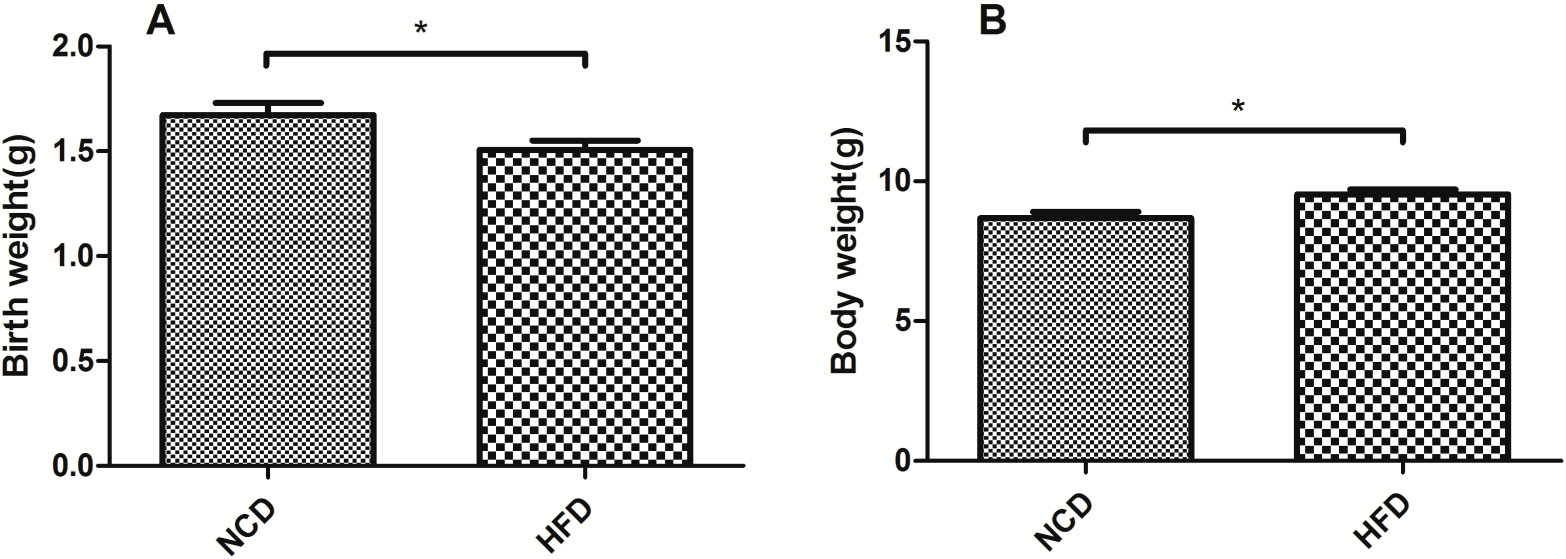
Birth weight and body weight at weaning in the offspring from the high-fat diet and normal chow diet groups. (**A**) Birth weight; and (**B**) body weight. Data represented as the mean ± standard deviation (S.D.) (*n* = 16, per group,8 male and 8 female). ^*^ *p* < 0.05 *vs.* the normal chow diet group. HFD, high-fat diet; NCD, normal chow diet.

The offspring of the dams fed a high-fat diet had a lower birth weight (*p* < 0.05, Figure 1A). However, after three weeks, the offspring had a higher body weight compared with the normal chow diet group at weaning (*p* < 0.05, Figure 1B). During this period, the calorie intake of the dams was not significantly different between the high-fat diet and normal chow diet groups.

### 2.2. Offspring of the Dams Fed a High-Fat Diet Exhibited Impaired Glucose Tolerance and Decreased Insulin Sensitivity

**Figure 2:**
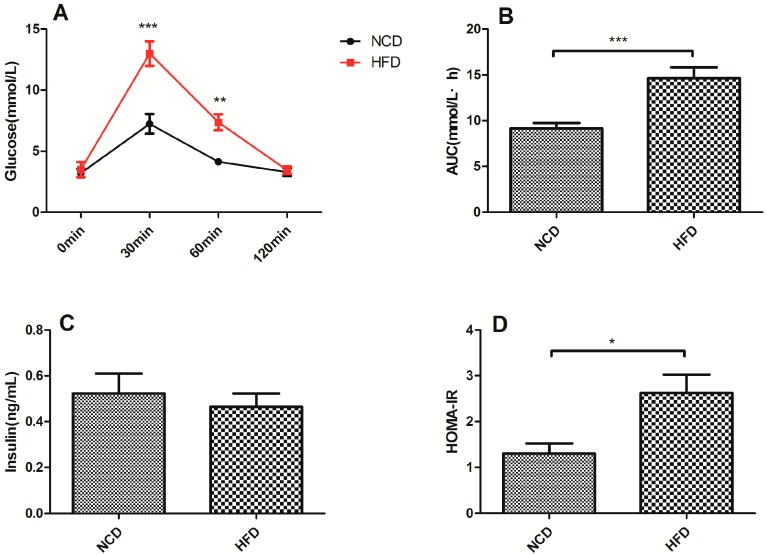
Glucose metabolism parameters of the offspring at weaning in the high-fat diet and normal chow diet groups. (**A**) IPGTT; (**B**) AUC; (**C**) Serum insulin levels; (**D**) HOMA-IR. Data represented as the mean ± S.D. (*n* =16, per group,8 male and 8 female). ^*^ *p* < 0.05, ^**^ *p* < 0.01, ^***^ *p* < 0.001 *vs.* the normal chow diet group. HFD, high-fat diet; NCD, normal chow diet; IPGTT, intraperitoneal glucose tolerance test; AUC, area under the curve; HOMA-IR, the homeostasis model assessment of insulin resistance.

**Figure 3:**
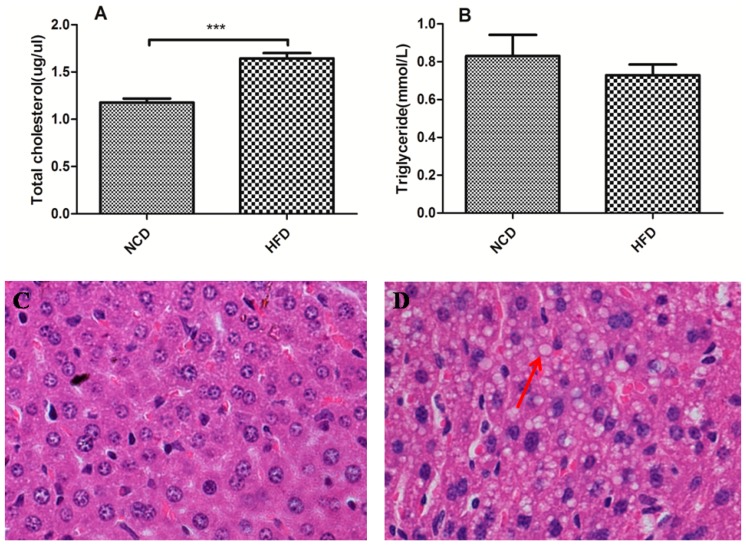
Lipid metabolism parameters of the offspring at weaning in the high-fat diet and normal chow diet groups. (**A**) Serum triacyglycerol and (**B**) total cholesterol; (**C**) Liver histology of haematoxylin and eosin staining in the offspring from the dams fed a normal chow diet had normal liver structure; (**D**) Lipid vacuoles (red arrow) of various sizes were observed within the hepatocytes of the offspring from the dams fed a high-fat diet. Original magnification, 40×. Data represented as the mean ± S.D. (*n* =16, per group,8 male and 8 female). ^***^ *p* < 0.001 *vs.* the normal chow diet group. HFD, high-fat diet; NCD, normal chow diet.

The blood glucose levels of the offspring of the dams fed a high-fat diet at weaning were significantly higher at 30 min (*p* < 0.001) and 60 min (*p* < 0.01) after intraperitoneal glucose administration (Figure 2A). Furthermore, as shown in Figure 2B, the area under the curve (AUC) was significantly larger for the high-fat diet group (*p* < 0.001, Figure 2C). To determine whether the high-fat diet reduced insulin sensitivity in the offspring, the levels of serum insulin were determined. There was no significant difference between the two groups (Figure 2D). However, the homeostasis model assessment of insulin resistance (HOMA-IR) was significantly higher in the high-fat diet offspring (*p* < 0.05, Figure 3B).

### 2.3. Offspring of the Dams Fed a High-Fat Diet Modulated Lipid Homeostasis

We determined that the total cholesterol was significantly elevated in the offspring of the dams fed a high-fat diet during pregnancy and lactation (*p* < 0.001, Figure 3A). However, no significant difference was detected of the serum triacylglycerol between the two groups (Figure 3B). However, there was no significant difference between HFD (high-fat diet) and NCD (normal chow diet) groups in female offspring (Table 1). Histological examination of the liver showed that the offspring of the dams fed a normal chow diet had normal liver structure at weaning (Figure 3C). However, we only observed lipid vacuoles of various sizes within the hepatocytes of the male offspring of the dams fed a high-fat diet at weaning (Figure 3D).

### 2.4. The Comparison of Biochemical Parameters of the Male and Female Offspring at Weaning

Furthermore, the metabolic parameters of the male and female offspring at weaning were compared between the high-fat diet and normal chow diet groups. It is indicated that body weight, AUC of intraperitoneal glucose tolerance test (IPGTT) and serum total cholesterol were significantly higher in male offspring of HFD group than NCD group. However, there was no significant difference between HFD and NCD groups in female offspring of these metabolic parameters (Table 1).

**Table 1:** The comparison of biochemical parameters of the male and female offspring at weaning in the high-fat diet and normal chow diet groups. Data represented as the mean ± S.D. (*n* = 8 per group of male and female). ^*^ *p* < 0.05, ^**^ *p* < 0.01, ^***^ *p* < 0.001 *vs.* the normal chow diet group.

Biochemical Parameters	Male			Female		
	NCD	HFD	*p* Value	NCD	HFD	*p* Value
FBG (mmol/L)	3.89 ± 1.56	5.19 ± 3.17	0.21	3.54 ± 1.72	4.89 ± 2.96	0.18
Body weight (g)	8.74 ± 1.14	9.73 ± 0.79	0.038 ^*^	8.79 ± 1.42	9.33 ± 0.53	0.32
AUC of IPGTT (mmol/L·h)	8.08 ± 1.49	16.45 ± 5.95	0.0016 ^**^	10.24 ± 2.69	12.97 ± 4.03	0.12
Serum insulin (µU/mL)	0.56 ± 0.37	0.37 ± 0.20	0.55	0.69 ± 0.19	0.51 ± 0.25	0.35
Triglyceride (mmol/L)	1.11 ± 0.43	0.83 ± 0.19	0.15	1.10 ± 0.71	0.62 ± 0.18	0.11
Total cholesterol (µg/uL)	1.24 ± 0.12	1.77 ± 0.21	<0.0001 ^***^	1.12 ± 0.17	1.22 ± 0.13	0.054

### 2.5. Maternal High-Fat Diet Regulated Gene Expression in the Male Offspring

**Figure 4:**
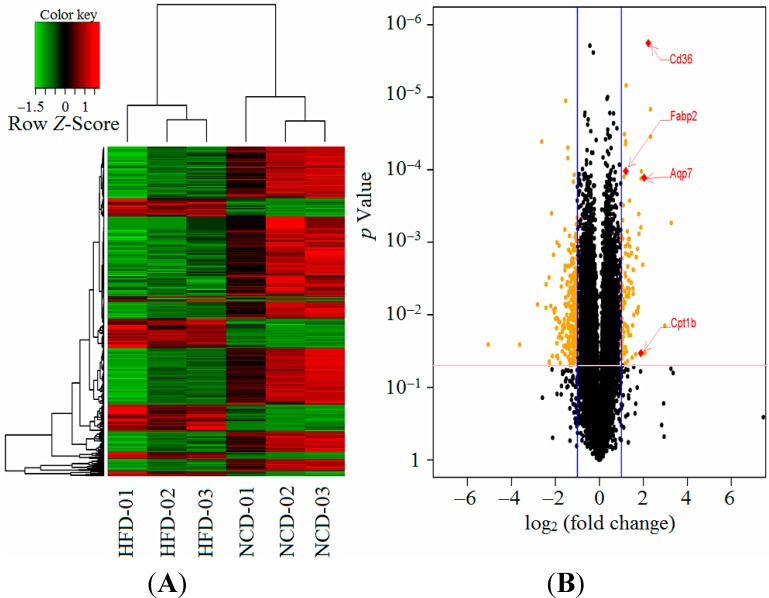
Gene array data analysis of the offspring between the high-fat diet and normal chow diet groups. (**A**) Heatmap diagram: this diagram illustrates the differential expression of hepatic mRNAs in the high-fat diet (*n* = 3) compared with the normal chow diet offspring (*n* = 3). The tree was based on the log_2_ transformation of the normalised probe signal intensity using hierarchical clustering. The hierarchical clustering was based on 380 differentially expressed mRNAs; the expression levels were differentially expressed in the high-fat diet group, with red representing increased expression and green representing decreased expression; (**B**) The Volcano Plot graphs: this graph shows the log_2_ of the fold change in each gene’s expression between the two group and its *p* value from the *t*-test. The blue lines indicate that the fold change in the gene expression threshold is 2. The pink line indicates that the *p* value of the *t*-test threshold is 0.05. There were four genes, which showed significantly different expression between the two groups, in the peroxisome proliferator-activated receptor (PPAR) signaling pathway. Aqp7, aquaporin 7; Cpt1b, carnitine palmitoyltransferase 1b; Fabp2, fatty acid binding protein 2; HFD, high-fat diet; NCD, normal chow diet.

The differentially expressed genes between the offspring at weaning were identified. There were 380 differentially expressed genes identified in the liver tissues between the two groups. One hundred and four genes (27.4%) were expressed at increased levels, whereas 276 genes (72.6%) were down-regulated in the high-fat diet group. Hierarchical clustering based on the similarity in gene expression using all differentially expressed genes highlighted the difference in the transcriptional profiles between the high-fat diet and normal chow diet groups (Figure 4A). The WebGestalt analysis of all differentially expressed genes in the high-fat diet group yielded 19 GO (Gene ontology)-categories (Table 2). The WebGestalt annotation tool was used for the identification of the putative KEGG (Kyoto Encyclopedia of Genes and Genomes) pathways. The genes were mapped to nine pathways (*p* < 0.05, Table 3).

The most common type of enriched pathway was related to the “Cell cycle” (*p* = 0.0001). The second and third most abundant pathways were related to the “Biosynthesis of unsaturated fatty acids” (*p* = 0.0044) and the “peroxisome proliferator-activated receptor (PPAR) signaling pathway” (*p* = 0.0125), respectively. The other pathways included the “Cytokine–cytokine receptor interaction” (*p* = 0.0232), “Glutathione metabolism” (*p* = 0.0232), “Inositol phosphate metabolism” (*p* = 0.0232), “Adipocytokine signaling pathway” (*p* = 0.0311), “Nitrogen metabolism” (*p* = 0.0311), and “Phosphatidylinositol signaling system” (*p* = 0.0369). In particular, we focused on the “PPAR signaling pathway” because PPARs have emerged as central regulators of glucose and lipid homeostasis. Four genes in this pathway, including Cd36, Aqp7 (aquaporin 7), Cpt1b (carnitine palmitoyltransferase 1b), and Fabp2 (fatty acid binding protein 2), were significantly differentially expressed on the gene array. The expression ratios are presented as the log_2_ (fold change) (Figure 4B). All four genes were up-regulated in this pathway.

### 2.6. Hepatic Gene Expression for the Validation of Array Analysis

We used qRT-PCR assays to verify some genes identified from our array results. Cd36, Aqp7, Cpt1b, and Fabp2 were selected for verification because of their true positive expression differences in the “PPAR signaling pathway” between the two groups, which is an essential pathway of glucose and lipid metabolism. All of them were significantly up-regulated in the high-fat diet group. A strong consistency between the array and qRT-PCR results was observed for all four genes, which indicated the reliability of our array assays. Furthermore, we examined expression of the PPAR isoforms alpha, gamma and delta, which may likely to explain the regulation of the PPAR pathway genes that we found. It is indicated that PPAR alpha and gamma increased significantly in the HFD group (*p* = 0.038 and *p* = 0.012, respectively. However, the expression of PPAR delta is very low, which even cannot be detected in some samples (Table 4).

**Table 2:** Gene ontology (GO) groups with differentially expressed genes in the high-fat diet offspring (*p* < 0.05).

GO Classification	GO Term	GO ID	*C*	*O*	*E*	*R*	Raw*p*	Adj*p*
Biological process	Response to pheromone	GO:0019236	101	9	1.3	6.93	6.37 ×10^−6^	0.0075
Biological process	G-protein coupled receptor signaling pathway	GO:0007186	2041	48	26.26	1.83	3.32 ×10^−5^	0.0196
Biological process	Response to chemical stimulus	GO:0042221	2194	49	28.23	1.74	9.92 ×10^−5^	0.0391
Biological process	Response to organic substance	GO:0010033	1538	37	19.79	1.87	0.0002	0.0394
Biological process	Response to stimulus	GO:0050896	7009	119	90.19	1.32	0.0002	0.0394
Biological process	Very long-chain fatty acid metabolic process	GO:0000038	24	4	0.31	12.95	0.0002	0.0394
Biological process	Regulation of mRNA processing	GO:0050684	46	5	0.59	8.45	0.0003	0.0443
Molecular function	Signal transducer activity	GO:0004871	2351	56	29.57	1.89	2.10 ×10^−6^	0.0001
Molecular function	Molecular transducer activity	GO:0060089	2351	56	29.57	1.89	2.10 ×10^−6^	0.0001
Molecular function	Pheromone binding	GO:0005550	91	9	1.14	7.86	2.22 ×10^−6^	0.0001
Molecular function	Pheromone receptor activity	GO:0016503	99	9	1.25	7.23	4.49 ×10^−6^	0.0001
Molecular function	Signaling receptor activity	GO:0038023	2127	52	26.76	1.94	2.47 ×10^−6^	0.0001
Molecular function	Transmembrane signaling receptor activity	GO:0004888	2039	49	25.65	1.91	8.11 ×10^−6^	0.0002
Molecular function	G-protein coupled receptor activity	GO:0004930	1612	37	20.28	1.82	0.0003	0.0063
Molecular function	Glutathione transferase activity	GO:0004364	26	4	0.33	12.23	0.0003	0.0063
Molecular function	Palmitoyl-CoA hydrolase activity	GO:0016290	15	3	0.19	15.9	0.0008	0.0153
Molecular function	Acyl-CoA hydrolase activity	GO:0047617	19	3	0.24	12.55	0.0016	0.0283
Molecular function	CoA hydrolase activity	GO:0016289	22	3	0.28	10.84	0.0025	0.0411
Molecular function	Carboxylesterase activity	GO:0004091	48	4	0.6	6.62	0.0031	0.0475

*C*, the number of reference genes in the category; *O*, the number of genes in the gene set and category; *E*, the expected number in the category; *R*, ratio of enrichment; Raw*p*, *p* value from the hypergeometric test; Adj*p*, *p* value adjusted by the multiple test adjustment.

**Table 3:** Kyoto Encyclopedia of Genes and Genomes (KEGG) pathways with differentially expressed genes in the high-fat diet offspring (fold change > 2.0, *p* < 0.05).

KEGG ID	Pathway Name	Gene Symbol	*C*	*O*	*E*	*R*	Raw*p*	Adj*p*
04110	Cell cycle	*Cdkn1a*, *Ccnb1*, *Mad2l1*, *Ccnb2*, *Gm5593*, *Wee1*, *LOC100045924*	127	7	0.8	8.74	2 × 10^−5^	0.0003
01040	Biosynthesis of unsaturated fatty acids	*Acot4*, *Acot3*, *Acot2*	25	3	0.16	19.02	0.0005	0.0044
03320	PPAR signaling pathway	*Aqp7*, *Fabp2*, *Cpt1b*, *Cd36*	80	4	0.5	7.93	0.0017	0.0125
04060	Cytokine-cytokine receptor interaction	*Il6ra*, *Il1r1*, *Il1r1*, *Cxcl13*, *Ccl20*, *Il22ra1*	245	6	1.55	3.88	0.0049	0.0232
00480	Glutathione metabolism	*Gsta2*, *Gsta1*, *Mgst3*	54	3	0.34	8.81	0.0049	0.0232
00562	Inositol phosphate metabolism	*Pten*, *Pik3c2g*, *Inpp4a*	57	3	0.36	8.34	0.0057	0.0232
04920	Adipocytokine signaling pathway	*Cpt1b*, *Ppargc1a*, *Cd36*	68	3	0.43	6.99	0.0092	0.0311
04070	Phosphatidylinositol signaling system	*Pten*, *Pik3c2g*, *Inpp4a*	78	3	0.49	6.1	0.0134	0.0369

*C*, the number of reference genes in the category; *O*, the number of genes in the gene set and also in the category; *E*, the expected number in the category; *R*, ratio of enrichment; Raw*p*, *p* value from the hypergeometric test; Adj*p*, *p* value adjusted by the multiple test adjustment.

**Table 4:** Fold change of gene expression measured by gene array and qRT-PCR. Aqp7, aquaporin 7; Cpt1b, carnitine palmitoyltransferase 1b; Fabp2, fatty acid binding protein 2.

Gene Symbol	Fold Change (Gene Array)	*p* Value (Gene Array)	Fold Change (qRT-PCR)	*p* Value (qRT-PCR)
*Cd36*	4.674	1.8048 × 10^−6^	4.9 ± 0.12	<0.0001
*Aqp7*	4.104	0.0001288	3.8 ± 0.27	0.0044
*Cpt1b*	3.705	0.03375443	3.7 ± 0.05	0.0292
*Fabp2*	2.302	0.00010463	2.5 ± 0.18	0.0002
*PPARα*	1.318	0.04499	1.32 ± 0.21	0.038
*PPARγ*	1.562	0.0041	1.61 ± 0.18	0.012

## 3. Discussion

The nutrition during several critical periods of early life, such as gestation and/or lactation, has significant influences on development and growth. Furthermore, maternal nutrition modification during pregnancy and/or lactation has been shown to influence health in the adult life of offspring, which can alter their responses to environmental challenges, and thus increase their predisposition to disease [12,13]. In the present study, we examined in a mouse model the consequences to the offspring of a maternal high-fat dietary regimen limited to the pregnancy and lactation periods. The findings indicate that short term maternal HF feeding can contribute to changes in the early life of the offspring’s physiology, including a lower birth weight, increased body weight, impaired glucose tolerance, insulin resistance and increased circulating total cholesterol levels compared with the normal chow diet group. The purpose and results of our study were distinct from some earlier animal experiments, which focused on the consequences for the offspring of maternal HF feeding during a substantially longer term HF dietary condition [3,14].

In the present study, the offspring of the dams fed a high-fat diet had a lower birth weight. However, after three weeks, the offspring had a higher body weight compared with the offspring of the dams fed a normal chow diet at weaning. The growth rate of the offspring indicates the phenomenon of catch-up growth. Increasing evidence suggests that rapid postnatal weight gain determines an increased susceptibility to obesity, insulin resistance, and diabetes mellitus [15]. These aberrant glucose and lipid metabolic conditions associated with catch-up growth are even severer when the catch-up growth operates during the early life stage, such as the lactation period [16]. Patterns of accelerated postnatal growth may be considered a physiological adaptation that allows the offspring to reach their genetically programmed weights [17]. One similar study indicated that within the first postnatal week pups exposed to maternal high-fat diet can consume more milk in tests of independent ingestion than controls [18]. Another study also suggested that pups cross-fostered to high-fat dams gained more body weight than chow pups by postnatal day 7, and persisted until weaning. Furthermore, postnatal high-fat pups had greater adiposity, higher plasma leptin concentration, impaired glucose tolerance, and reduced phosphorylated signal transducer and activator of transcription (STAT)3 in response to leptin in the arcuate nucleus at weaning [19]. In our study, it is demonstrated that the male offspring are more prone to develop abnormal glucose and lipid metabolism than female offspring. Similar phenomena were found in adulthood offspring [10,14]. This can be explained by the differential ingestive behavior and estrogen level between the male and female offspring due to lower food intake and higher estrogen level in female. A recent study found sex differences in pancreatic β-cell function and suggested that oxidative stress may play an important role in the sex differences observed [14].

In addition to the body mass and specific serum markers of metabolic syndrome that have been examined in mice born to mothers given a high-fat diet during pregnancy and lactation, a substantial number of lipid vacuoles within the hepatocytes were observed in the high-fat diet offspring, but not the normal chow diet offspring at weaning. A previous study in rats indicated that the offspring of high-fat fed dams during pregnancy and lactation had an increased liver weight and liver triacylglycerols content [20]. Another study in mice also demonstrated that the offspring of high-fat diet fed dams had profound metabolic defects. More specifically, the offspring developed non-alcoholic fatty liver disease and non-alcoholic steatohepatitis at 15 weeks of age. More severe forms of non-alcoholic fatty liver disease and non-alcoholic steatohepatitis developed at 30 weeks of age [9]. It has been reported in humans that hypercholesterolemia and non-alcoholic fatty liver disease were strongly associated with an increased risk of metabolic symdrome [21]. Therefore, our research suggested that a maternal high-fat diet can induce metabolic syndrome and hepatic steatosis in the early life stages of offspring. These findings may be utilized as convincing evidence for the prevention and intervention of metabolic diseases from the early stage of life.

In our gene array research, all differentially expressed genes in the high-fat diet group were mapped to nine pathways by WebGestalt and KEGG pathway analyses. The third most significant pathway was the “PPAR signaling pathway” (*p* = 0.0125), and qRT-PCR further verified these results. This study shows for the first time that a maternal high-fat diet only during the pregnancy and lactation periods leads to metabolic syndrome in the early life of the mice offspring through the PPAR signaling pathway. A previous study in rats also demonstrated that a maternal HF diet leads to alterations in PPAR gene expression in the offspring. However, the period of maternal HF diet was much longer, *i.e.*, the dams were fed the corresponding diet 6 weeks before mating and throughout gestation and lactation [22].

Peroxisome proliferator-activated receptors (PPARs) are ligand-activated transcription factors, which belong to the nuclear hormone receptor super-family. PPARs have in three isoforms: PPARα, PPARβ/Δ and PPARγ [23]. With regard to lipid and carbohydrate metabolism, PPARs have emerged as major regulators [24]. They are considered central regulators of glucose and lipid homeostasis. Furthermore, they are the molecular targets for drugs to treat hypertriglyceridaemia and type 2 diabetes mellitus [25]. In our study, it is indicated that PPARα and PPARγ increased significantly in the HFD group. Similarly, the offspring of suboptimal maternal nutrition were obese at weaning, and accompanied by elevated gene expression for PPARγ (PPARG) and its co-activator PGC1α [26]. A previous study in rats also demonstrated that maternal malnutrition leads to alterations in PPAR gene expression (PPARα and PPARγ) in the offspring at 18 months of age [27]. Another study indicated that short-term Pioglitazone (a potential role for drugs that activate PPARγ receptors) therapy in the offspring of obese mothers attenuates metabolic changes associated with the developmental programming of metabolic syndrome [28]. A recent study demonstrated that maternal diet induced obesity contributed to metabolism disorder, hepatic lipotoxicity, and liver steatosis in the offspring, and Bezafibrate, which targets PPAR, had beneficial effects in ameliorating these disorders, predominately through the PPARγ activation and the increased PPARα/PPARγ ratio in the liver [29]. Some synthetic PPAR ligands have been widely used in the treatment of dyslipidemia (e.g., fibrates–PPARα activators) and diabetes mellitus (e.g., thiazolidinediones–PPARγ agonists) [30,31]. New generation drugs, such as PPARα/γ dual agonists, prevent the development of obesity and reduce the lipid accumulation in cardiac cells, even during a high-fat diet [32]. These findings suggest that the PPAR signaling pathway may play an important role in regulating lipid and glucose homeostasis, and the PPARs and their modulators have been suggested for the treatment of metabolic disorders, such as hyperglycaemia and dyslipidemia.

## 4. Materials and Methods

### 4.1. Animals and Experimental Protocol

Seven-week-old C57BL/6J mice were obtained from the Institute of Laboratory Animal Science, Chinese Academy of Medical Sciences and Peking Union Medical College (Beijing, China; RSCXK-2013-0107). The animals were maintained under controlled conditions (room temperature at 22 ± 2 °C; 12 h light/dark cycle) and fed a normal chow diet. After one week for acclimatization after arrival, the dams were mated to the high-fat diet fed C57Bl/6J males. The females were checked daily for postcopulatory plugs, and the presence of a plug in the morning after mating was considered d 0.5 of pregnancy. Then, the pregnant mice were randomly assigned to two groups and were fed a high-fat diet or normal chow diet. The high-fat diet contained (kcal %): fat, 58%; carbohydrate, 25.6%; and protein, 16.4% and the energy density was 23.4 kJ/g, whereas the normal chow diet was containing (kcal %): fat, 11.4%; carbohydrate, 62.8%; and protein, 25.8%. and the energy density was 12.6 kJ/g, as previous described [33]. The dames were fed a high-fat diet or normal chow diet during pregnancy and lactation. They had free access to food and water, and their daily food consumption was estimated by weighing the remaining food. The litter sizes were standardized to 6 pups as 3 males and 3 females to ensure no litter was nutritionally biased. All offspring were weaned at 3 weeks of age. At weaning, the mice (*n* = 16, 8 male and 8 female per group, one male and female offspring per litter) were sacrificed. Blood samples were taken from the intraorbital retrobulbar plexus after 10-h of fasting in anesthetised mice, and the liver samples were quickly removed, snap frozen in liquid nitrogen, and stored at −80 °C for further analysis. All surgeries were performed under chloral hydrate anaesthesia, and all efforts were made to minimize suffering. The animal care and use committee of the Peking Union Medical College Hospital (Beijing, China, MC-07-6004, 3 May, 2013) approved all the procedures. All the operations involving animals were conducted according to the Guide for the Care and Use of Laboratory Animals [34].

### 4.2. Measurement of Body Weight

The maternal weights on day 0.5 and day 20 of gestation and the neonatal weights on day 0.5 after birth were measured. The offspring’s body weights were then measured from birth to weaning twice per week for each mouse.

### 4.3. Glucose Tolerance Tests

After the mice had fasted for 10-h, glucose (2.0 g/kg body weight) was intraperitoneally administered. BG (Blood Glucose) levels were measured before the injection (time 0) and at 30, 60, and 120 min after the injection using a Contour TS glucometer (Bayer, Beijing, China) with blood from a tail bleed. The area under the curve (AUC) of intraperitoneal glucose tolerance test (IPGTT) was calculated by the trapezoid formula: AUC = 0.5 × (BG0 + BG30)/2 + 0.5 × (BG30 + BG60)/2 + 1 × (BG60 + BG120)/2 [35].

### 4.4. Measurement of Serum Insulin, Triacylglycerol and Total Cholesterol Levels

At 3 weeks of age, the offspring mice were euthanized, and the blood samples were collected. The blood samples were centrifuged at 4000× *g* for 10 min, and the serum was stored in aliquots at −80 °C. The serum insulin concentrations were measured using the Mouse Ultrasensitive Insulin ELISA kit from ALPCO Diagnostics (80-INSMSU-E01, Salem, NH, USA). The intra-assay coefficients of variation for the insulin measurements were 4.2%. The insulin sensitivity was assessed using the homeostasis model assessment of insulin resistance (HOMA-IR). The HOMA-IR was calculated as the fasting insulin concentration (μU/mL) × fasting glucose concentration (mmol/L)/22.5 [36]. The serum cholesterol (K603-100, kits from BioVision Inc., Mountain View, CA, USA) and triacylglycerol (K622-100, kits from BioVision Inc.) were measured by colorimetric methods.

### 4.5. Liver Histological Analysis

The liver tissues were fixed with formaldehyde solution and embedded in paraffin. Then, 8 μm sections were cut using a microtome and mounted on glass slides. Deparaffinized and fixed sections were stained with haematoxylin and eosin. Microscopic examination was performed on stained liver sections from representative offspring mice in each group.

### 4.6. RNA Preparation and Gene Array Experiments

Because there was no significant difference between HFD and NCD groups in female offspring. Liver tissues of male offspring mice to perform the array experiments. Total RNA from the liver tissues was extracted using TRIzol reagent (Life Technologies Inc., Carlsbad, CA, USA), according to the manufacturer’s instructions. The concentrations of total RNA were measured using a Nanodrop (ND-1000, NanoDrop products, Bancroft Building, Wilmington, DE, USA). All samples had high quality and showed no signs of DNA contamination or RNA degradation. The RNA samples were immediately frozen and stored at −80 °C until further analysis. The gene expression profiles in the liver tissues from the mice were determined using whole genome-wide gene expression array analyses. Because of financial constraints, we could not perform a whole genome array for each biological replicate. Thus, each group contained three biological replicates, which can obtain a relatively reliable estimate of the mean gene expression. Therefore, a total of six gene arrays were analyzed. The samples were processed following Affymetrix recommendations, and the cDNA was hybridized to the Affymetrix Mouse Gene ST 1.0 array (Affymetrix, Santa Clara, CA, USA). Both the background correction and normalization were done using a PLIER (Probe Logarithmic Intensity Error) algorithm. *t*-test was used for pre-processing and statistical analysis. The fold change and *p*-value were used to identify the probe sets that showed significant differential expression between the experimental conditions. Specifically, the probes with a |fold change| >2 and a significant difference (*p* < 0.05) were chosen for further analysis. Differentially expressed genes were hierarchically clustered using *g*-plots *R* package.

### 4.7. Gene Array Data Analysis

The signals were averaged for liver tissues from the high-fat diet and normal chow diet groups, and the fold changes were calculated based on the average values from each group. The genes were selected as significant using a combined criterion for true positive expression differences of a |fold change| >2.0 and a *p*-value <0.05. Cluster analyses were done using the software of *g*-plots *R* package. To identify the biological significance of the group of genes with changed expression, the subset of genes that met the above criteria was analyzed with the GO (Gene Ontology) classification system and the KEGG (Kyoto Encyclopedia of Genes and Genomes) pathways using the web-based tool WebGestalt (WEB-based GEne SeT AnaLysis Toolkit) [37]. The over-representation of genes with altered expression within specific GO categories was determined through a hypergeometric test [38].

### 4.8. Quantitative RT-PCR Experiment

For validation of the array results, four genes from the gene list were selected for qRT-PCR analysis by expanding the quantity samples in male offspring (*n* = 8 per group). Furthermore, we examined expression of the PPAR isoforms α, γ and ∆, which may likely to explain the regulation of the PPAR pathway genes that we found. Prior to PCR, each total RNA was processed with Rnase-free Dnase (Qiagen, New York, NY, USA). The RNA was reverse transcribed by 1 μg of total RNA from each sample using the Power cDNA Synthesis kit (A3500, Promega BioSciences LLC, Sunnyvale, CA, USA). The cDNA (2 μL) was amplified with a SYBR^®^ Green PCR Master Mix (RR420A, Takara Bio Inc., Otsu, Shiga, Japan) in a final volume of 20 μL. We used Oligo 7.0 software (Molecular Biology Insights, Inc., Cascade, CO, USA) to design the following sequences of the primers for *Cd36*, *Aqp7* (*aquaporin 7*), *Cpt1b* (*carnitine palmitoyltransferase 1b*), *Fabp2* (*fatty acid binding protein 2*), PPARα (*peroxisome proliferator activator receptor alpha*), PPARΔ (*peroxisome proliferator activator receptor delta*), PPARγ (peroxisome proliferator activator receptor gamma) and β-actin: *Cd36*, forward 5'-CAAGCTATTGCGACATGATT-3' reverse 5'-CGAACACAGCGTAGATAGAC-3'; *Aqp7*, forward 5'-CTCGTGATAGGCATCCTTGT-3' reverse 5'-AGCCAGCAATGAAAGTGAAC-3'; *Cpt1b*, forward 5'-TATTACCGCATGGAGACATT-3' reverse 5'-TTAGTTGCCCACCATGACTT-3'; *Fabp2*, forward 5'-GACAATGGAAAGGAGCTGAT-3' reverse 5'-CCAGGCTCTGAGAAGTTGAC-3'; *PPARα*, forward 5'-CTGTCCGCCACTTCGAGTC-3' reverse 5'-GATACGCCCAAATGCACCAC-3'; PPARΔ, forward 5'-TCACCAGCAGCCTAAAAGCA-3' reverse 5'-CCGGCGAGAAGAAAGGAAGT-3'; *PPARγ*, forward 5'-CGGGCTGAGAAGTCACGTT-3' reverse 5'-TGCGAGTGGTCTTCCATCAC-3'; *β-actin*, forward 5'-TGTTACCAACTGGGACGACA-3' reverse 5'-GGGGTGTTGAAGGTCTCAAA-3'. The reaction production was accurately measured in the exponential phase of amplification by the ABI prism Vii7 Sequence Detection System (ABI Prism^®^ Vii7, Applied Biosystems, Life Technologies). The reaction conditions consisted of an initial activation step (30 s at 95 °C) and a cycling step (denaturation for 5 s at 95 °C and annealing for 34 s at 60 °C for 40 cycles). All reactions were carried out with three biological replicates, and each analysis consisted of three technical replicates and a melting curve analysis that was performed after each run. The sequences of the primers used are listed in Table 1. The signal of the housekeeping gene β-actin was used for normalization, and the relative expression levels were quantified by the 2^−ΔΔ*Ct*^ method, with the relative fold changes normalized to the control values [39].

### 4.9. Statistical Analysis

The data were normally distributed and were expressed as the mean ± standard deviation (S.D.). Statistical analyses were performed with ANOVA followed by Student’s *t*-test. A *p* value <0.05 was considered statistically significant. All statistical analyses were calculated with SPSS 15.0 (SPSS Inc., Chicago, IL, USA).

## 5. Conclusions

In conclusion, our data suggest that a maternal high-fat diet during pregnancy and lactation predisposes the offspring to the development of obesity, glucose intolerance and dyslipidemia in the early stages of life. More importantly, the PPAR signaling pathway may be the underlying mechanism that accounts for the phenomenon, which indicates that maternal nutrition can impair glucose and lipid metabolism in the early life of the offspring. However, the long-term effects of these metabolic abnormalities must be further examined. Pregnancy and lactation are the pivotal periods of growth and development. It is expected to facilitate the future development of a novel drug for the prevention and intervention of metabolic diseases during the early stage of life.
